# A Unique Role for Nonmuscle Myosin Heavy Chain IIA in Regulation of Epithelial Apical Junctions

**DOI:** 10.1371/journal.pone.0000658

**Published:** 2007-08-01

**Authors:** Andrei I. Ivanov, Moshe Bachar, Brian A. Babbin, Robert S. Adelstein, Asma Nusrat, Charles A. Parkos

**Affiliations:** 1 Epithelial Pathobiology Research Unit, Department of Pathology and Laboratory Medicine, Emory University, Atlanta, Georgia, United States of America; 2 Laboratory of Molecular Cardiology, National Heart Lung and Blood Institute, National Institute of Health, Bethesda, Maryland, United States of America; Dresden University of Technology, Germany

## Abstract

The integrity and function of the epithelial barrier is dependent on the apical junctional complex (AJC) composed of tight and adherens junctions and regulated by the underlying actin filaments. A major F-actin motor, myosin II, was previously implicated in regulation of the AJC, however direct evidence of the involvement of myosin II in AJC dynamics are lacking and the molecular identity of the myosin II motor that regulates formation and disassembly of apical junctions in mammalian epithelia is unknown. We investigated the role of nonmuscle myosin II (NMMII) heavy chain isoforms, A, B, and C in regulation of epithelial AJC dynamics and function. Expression of the three NMMII isoforms was observed in model intestinal epithelial cell lines, where all isoforms accumulated within the perijunctional F-actin belt. siRNA-mediated downregulation of NMMIIA, but not NMMIIB or NMMIIC expression in SK-CO15 colonic epithelial cells resulted in profound changes of cell morphology and cell-cell adhesions. These changes included acquisition of a fibroblast-like cell shape, defective paracellular barrier, and substantial attenuation of the assembly and disassembly of both adherens and tight junctions. Impaired assembly of the AJC observed after NMMIIA knock-down involved dramatic disorganization of perijunctional actin filaments. These findings provide the first direct non-pharmacological evidence of myosin II-dependent regulation of AJC dynamics in mammalian epithelia and highlight a unique role of NMMIIA in junctional biogenesis.

## Introduction

The apical junctional complex (AJC) represents one of the most characteristic cellular structures of differentiated simple epithelia [1]–[3]. It is positioned at the apical-most aspect of the lateral plasma membrane and regulates the integrity of epithelial monolayers, formation of paracellular barrier and cell polarity [1]–[3]. The AJC is composed of two multiprotein complexes known as the tight junction (TJ), and adherens junction (AJ) [1]–[5]. These complexes are regulated by three major types of protein-protein interactions. The first type is homotypic interactions between transmembrane TJ/AJ proteins on the opposing cell plasma membranes. Such interactions have been attributed to the TJ components occludin and claudins [2]–[4], as well as to AJ proteins E-cadherin and nectins [6]–[8]. The second type of interactions involves a number of scaffolding proteins on the cytosolic side of the plasma membrane that cluster and stabilize transmembrane components of TJs and AJs and create so-called cytosolic junctional plaques [2], [5], [9]. The third type of interactions is mediated by actin-binding proteins such as members of “zonula occludens” (ZO) family, afadin, α-catenin and vinculin that may physically link the AJC to actin microfilaments [9]–[11].

In polarized epithelial cells, actin microfilaments are organized into a characteristic perijunctional belt positioned at the apical pole at the level of the AJC [12], [13]. The integrity of this F-actin belt is critical for maintenance of the AJC structure and functions [14], [15], whereas reorganization of cortical actin microfilaments drives TJ/AJ disassembly and reassembly during respectively loss and reestablishing of epithelial cell polarity [16]–[21]. How reorganization of F-actin regulates remodeling of epithelial junctions remains poorly understood. Several lines of evidence have implicated a key F-actin motor, nonmuscle myosin (NMM) II in the remodeling of epithelial apical junctions. For example, NMMII has been shown to regulate paracellular permeability in renal and intestinal epithelial cell monolayers [22]–[24]. In addition, disassembly and internalization of the AJC during extracellular calcium depletion [17] and interferon-γ treatment [25] has been shown to be driven by NMMII-dependent contraction of perijunctional F-actin belt and apical F-actin-coated vacuoles respectively. Finally, we and others have demonstrated that NMMII regulates the assembly of epithelial AJs and TJs as well as the establishment of apico-basal cell polarity [18], [21], [26], [27].

It is noteworthy, that NMMII has been implicated in the regulation of AJC dynamics in mammalian epithelia based primarily on the results of pharmacological inhibition studies. Early investigations used a nonselective inhibitor of conventional myosin, butanedione monoxime [28], [29], whereas results of most recent studies have been obtained using a more selective NMMII inhibitor, blebbistatin [18], [21], [26], [27]. However, butanedione monoxime does not inhibit NMMII [30], [31] and is therefore not useful in studies of myosin II in epithelial cells. Likewise, a recent report has revealed non-myosin-related cellular effects of blebbistatin [32]. Hence, results from previous studies employing pharmacological inhibition of NMMII should be interpreted with caution. Although genetic analysis has implicated myosin II in the remodeling of epithelial cell-cell adhesion during invertebrate embryonic morphogenesis [33], no genetic evidence is available to support the role of myosin II in junctional dynamics in differentiated mammalian epithelia.

NMMII functions as a heterohexamer composed by two heavy chains and two pairs of light chains [34], [35]. The heavy chain possesses enzymatic activity and utilizes ATP to drive actin filament movement [34], [35]. Three isoforms, A, B, and C, of mammalian NMMII have been identified to date [36], [37], that are widely expressed in different tissues and have 64–80% amino acid identity. Despite these similarities, they are not functionally redundant and appear to have different roles in cell motility, cytokinesis, regulation of cell shape and intracellular vesicular traffic [38]–[43].

Two recent studies have addressed the role of NMMII isoforms in the regulation of epithelial cell-cell adhesion. A study by one of us [44] demonstrated that genetic ablation or small interfering RNA (siRNA)-mediated knock-down of NMMIIA expression resulted in decreased adhesiveness of mouse embryonic stem cells which correlated with loss of AJ proteins, E-cadherin and β-catenin from intercellular contacts. Decreased accumulation of AJ proteins at cell-cell junctions has also been reported after siRNA-mediated knock-down of NMMIIB in COS-7 embryonic kidney epithelial cells [26]. However, since embryonic cells do not form TJs, and lack an apical actomyosin belt, these observations can not be simply extrapolated to differentiated adult epithelia. Furthermore, the expression pattern of NMMII isoforms demonstrates major differences between embryonic differentiated adult epithelial cells. In particular, NMMIIA and NMMIIC, which are abundant in differentiated epithelia are not expressed in COS-7 cells [38] and mouse embryonic stem cells [44] respectively.

Since the myosin II isoform (s) that regulate dynamics and functions of the AJC has not yet been identified, we investigated the roles of NMMIIA, NMMIIB, and NMMIIC in biogenesis of apical junctions in simple polarized epithelia. RNA interference in cultured intestinal epithelial cells was used in concert with a classic calcium switch model to examine which isoform of myosin II controls different steps of AJC reorganization including assembly of initial AJ-like junctions, formation of TJs, and disruption of apical junctions.

## Results

### Expression and localization of NMMII isoforms in human intestinal epithelial cells

**Figure 1:**
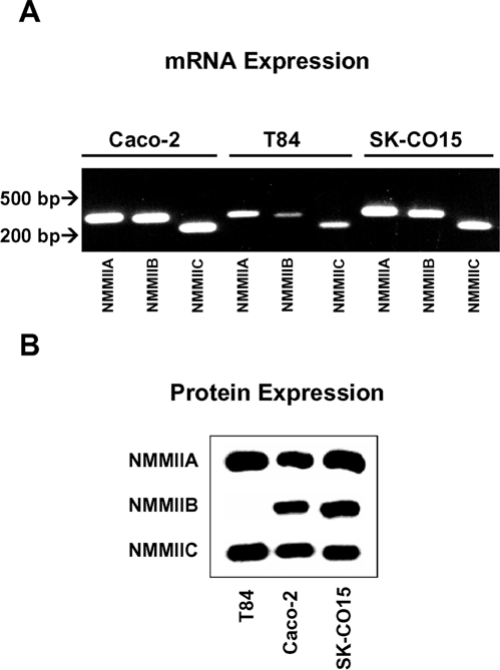
Expression of nonmuscle myosin II isoforms in cultured human intestinal epithelial cells. mRNA (A) and protein (B) expression of NMMIIA, NMMIIB and NMMIIC was analyzed in different intestinal epithelial cell lines using isoform-specific primers and polyclonal antibodies. NMMIIA and NMMIIC are expresses in all studied epithelial cells lines, whereas protein expression of NMMIIB is abundant in SK-CO15 and Caco-2 cells but is undetectable by Western blotting in T84 colonic epithelial cells.

In order to examine roles of different myosin II heavy chain isoforms in the regulation of AJC in simple polarized epithelia, we used SK-CO15, Caco-2 and T84 human colonic epithelial cell lines. These cells form high-electrical resistance (400–2,000 Ohm × cm^2^) monolayers that possess well defined TJs and AJs [17], [45]–[47]. We first analyzed the expression of NMMIIA, NMMIIB, and NMMIIC in these cell lines by RT-PCR and Western blotting. RT-PCR analysis with isoform-specific primers revealed abundant mRNA expression of all three isoforms in Caco-2 and SK-CO15 cells (Figure 1A). Strong signals from NMMIIA and NMMIIC mRNAs were also detected in T84 cells, whereas the expression of NMMIIB message in these cells was weak (Figure 1A). Western blotting analysis revealed strong protein expression of NMMIIA and NMMIIC protein in three epithelial cell lines as well as abundant expression of NMMIIB in Caco-2 and SK-CO15 cells (Figure 1B). In contrast, NMMIIB protein was not observed by Western blotting analysis of T84 total cell lysates (Figure 1B), and was detected in these cells only after immunoprecipitation with anti NMMIIB antibody (data not shown). These results demonstrate that well differentiated colonic epithelial cells coexpress all NMMII isoforms, although relative expression of different NMMII heavy chains is likely to be variable depending on cell type.

**Figure 2:**
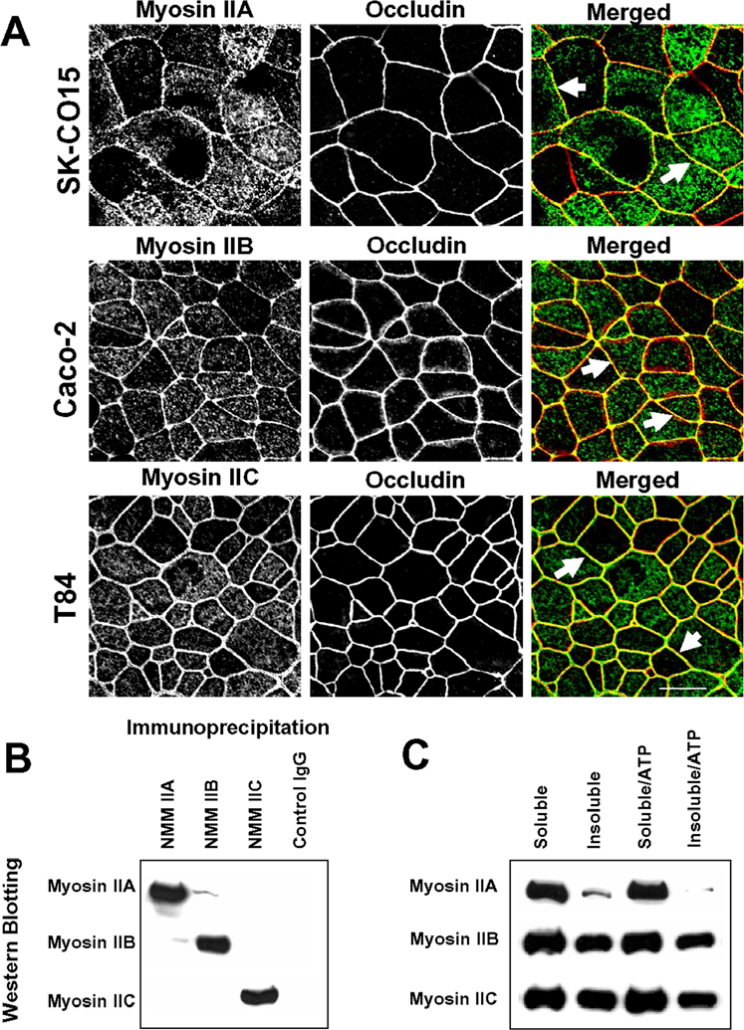
Localization and biochemical properties of different NMMII isoforms in cultured human intestinal epithelial cells. (A) Confluent SK-CO15, Caco-2 and T84 cell monolayers were double-immunolabeled for either NMMIIA, NMMIIB, or NMMIIC (green) and the TJ protein, occludin (red). All three NMMII isoforms colocalize with occludin at the mature AJC (arrows). Bar, 10 µm. (B). NMMIIA, NMMIIB, and NMMIIC were immunoprecipitated from SK-CO15 cell lysates using isoform-specific polyclonal antibodies. Little or no cross-precipitation of the different NMMII isoforms is observed. (C) Detergent solubility of different NMMII isoforms was analyzed using TX-100 fractionation of SK-CO15 cell monolayers. The majority of NMMIIA is Triton-soluble, especially in the presence of 1 mM ATP, whereas significant Triton-insoluble fraction is characteristics to NMMIIB and NMMIIC.

We next examined which myosin II isoform(s) localize to the AJC in polarized intestinal epithelium. Double immunolabeling and confocal microscopy of confluent SK-CO15, Caco-2 and T84 monolayers revealed significant enrichment of NMMIIA, NMMIIB, and NMMIIC at the perijunctional actomyosin belt and their colocalization with the TJ protein occludin (Figure 2A arrows). Given the similar localization pattern observed for the three NMMII isoforms and the fact that other conventional myosin heavy chains form heterodimers [48], we next determined whether different NMMII isoforms can form mixed dimers in intestinal epithelial cells. Immunoprecipitation experiments were performed using NMMII isoform-specific antibodies, all of which (but not the IgG control) effectively precipitated designated myosin II isoforms from SK-CO15 cell lysates (Figure 2B). However, only trace amounts of NMMIIB were detected in immunoprecipitates obtained with the NMMIIA antibody and *vice versa* (Figure 2B). Furthermore, neither NMMIIA, nor NMMIIB antibodies precipitated NMMIIC, and no NMMIIA or NMMIIB was detected in NMMIIC immonoprecipitates (Figure 2B). This data suggest that different NMMII isoforms do not form heterodimers in human colonic epithelial cells.

Since myosin II functions are dependent on physical interactions with F-actin, we compared the actin-binding ability of different NMMII isoforms in epithelial cells. We used solubility in Triton X (TX)-100-containing buffer as a qualitative measure of the strength of NMMII binding to actin microfilaments. As shown in Figure 2C, the majority (89±11%; n = 3) of NMMIIA in SK-CO15 cells was TX-100-soluble, and the relative amount of insoluble fraction was decreased in the presence of 1 mM ATP. The amounts of TX-100-soluble NMMIIB and NMMIIC (64±5%; n = 3, and 54±6%; n = 3, respectively) were significantly lower than values obtained for NMMIIA (p<0.05). In addition, TX-100 solubility of NMMIIB and NMMIIC was not substantially changed by addition of ATP (Figure 2C). This data suggests more labile association of NMMIIA with actin filaments compared to the other myosin II isoforms.

### siRNA-mediated downregulation of NMMIIA expression altered epithelial cell shape and attenuated development of the paracellular barrier

**Figure 3:**
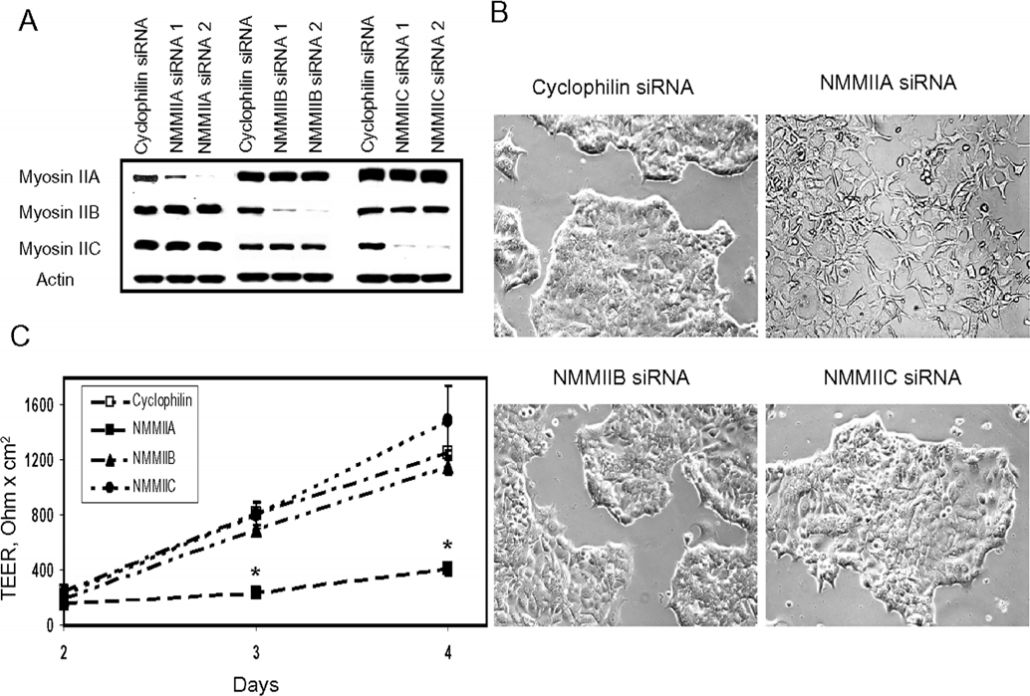
Downregulation of the NMMIIA expression alters epithelial cell shape and attenuates development of the paracellular barrier. (A) Western blots of SK-CO15 cell lysates prepared 3 days after transfection show selective downregulation of protein expression of NMMIIA. NMMIIB, and NMMIIC by two different siRNA duplexes, each specific for the NMMII isoform. (B) siRNA-mediated knock-down of NMMIIA but not NMMIIB or NMMIIC causes dramatic changes from an orthogonal epithelial to a protrusive fibroblast-like shape in low-density colonies of SK-CO15 cells. (C) siRNA -mediated knock-down of NMMIIA but not NMMIIB or NMMIIC significantly attenuates the increase in TEER in confluent SK-CO15 cell monolayers when compared to the control (cyclophilin) siRNA-transfected cells (*p<0.05; n = 4).

To gain insight into functions of different myosin II isoforms in epithelia, we used RNA interference technology to selectively down-regulate expression of NMMIIA, NMMIIB and NMMIIC in SK-CO15 cells. As shown in Figure 3A, by using two different siRNA sequences for each target, we dramatically decreased expression of NMMIIA, NMMIIB and NMMIIC without significant effects on expression of the other isoforms. All subsequent data presented in this paper were obtained using siRNA duplexes #2 for NMMIIA, NMMIIB and NMMIIC (Table 1). Densitometric analysis of Western blots revealed that transfection with these duplexes reduced protein levels of NMMIIA, NMMIIB and NMMIIC by approximately 96, 98 and 99% respectively. All major effects on cell morphology and AJC dynamics were reproducible when alternative siRNA duplexes presented in Table 1 or isoform-specific siRNA SmartPools were used (data not shown).

**Table 1:** Sequences of PCR primers and siRNA duplexes used to analyze and downregulate expression of individual NMMII isoforms.

Myosin II isoform	NMMIIA	NMMIIB	NMMIIC
PCR primers	Forward: 5′-ggccgaagaggaggcccag	Forward: 5′-cgacgcgtgccaacgcatc	Forward: 5′-ctcctctagtcggaagacctggc
	Reverse: 5-cggcaggtttggcctcag	Reverse: 5′-gacacagttgatctttcaggaagg	Reverse: 5′-ctgcccttgagtctaagttgg
siRNA duplex 1	5′-gcacagagcuggccgacaauu	5′-ucagaaaccucgacaauuauu	5′-gaacggaacaccgaucaaguu
siRNA duplex 2	5′-ggccaaaccugccgaauaauu	5′-gaaugaagcuuccguuuuauu	5′-cuucggagcuucacggguuuu

In assessing effects of NMMIIA knock-down on SK-CO15 cells, we noticed dramatic alterations in cell shape. When cells grown at low (20–30%) density were transfected with NMMIIA-specific siRNA, they acquired a fibroblast-like shape characterized by long peripheral protrusions (Figure 3B), in contrast to control cells and ones with downregulated NMMIIB and NMMIIC, which formed tightly packed colonies of orthogonally-shaped cells (Figure 3B). Such effect on the cell shape did not appear to be cell-line specific, since siRNA-mediated depletion of NMMIIA caused peripheral protrusions in Caco-2 cells and HT-1080 fibrosarcoma cells (data not shown). In addition, similar changes of cell shape were previously observed after overexpression of a truncated C-terminal fragment of NMMIIA in HeLa cells [43] as well as after pharmacological inhibition of myosin II with blebbistatin in SK-CO15, Caco-2 epithelial cells (data not shown) and NIH 3T3 fibroblasts [49]. These results suggest that the observed morphological changes are a general consequence of interfering with NMMIIA function.

Since acquisition of a protrusive fibroblast-like shape has been shown to correlate with decreased epithelial cell-cell adhesion [47], [50], we investigated the effects of downregulation of myosin II on epithelial barrier. SK-CO15 cells were cultured at a high (50–60%) density on permeable filters and transfected with either control (cyclophilin B) or the NMMII isoform-specific siRNAs. Development of barrier function in transfectants was monitored by transepithelial electrical resistance (TEER) measurement. As shown in Figure 3C, control cells developed TEER values in the range of 1,200 Ohm × cm^2^ on a day 4 post-transfection. The development of TEER in cells deficient in either NMMIIB or NMMIIC paralleled those of the control monolayers. In contrast, NMMIIA-depleted monolayers demonstrated a significant delay in the development of TEER only reaching values in the range of 300 Ohm × cm^2^ (Figure 3C). We also investigated the integrity of AJs and TJs in cells lacking different NMMII isoforms by confocal microscopy after immunolabeling of different AJ/TJ proteins. On day 4 post-trasfection, we observed identical localization of E-cadherin (Figure S1, arrows) and occludin (Figure S2, arrows) at the intercellular contacts in confluent control, NMMIIA, NMMIIB and NMMIIC-depleted cell monolayers. Furthermore, Western blotting analysis demonstrated that downregulation of NMMIIA or other myosin II isoforms did not decrease the expression of major AJ (E-cadherin, β-catenin, p120-catenin) and TJ (occludin, cingulin, afadin, ZO-1, claudin-4) proteins (Figure S3). This data indicate that NMMIIB and NMMIIC-depleted SK-CO15 cells were able to form structurally and functionally normal AJs and TJs, whereas NMMIIA-deficient cells formed structurally–normal, yet functionally defective AJC.

### siRNA-mediated knock-down of NMMIIA attenuated reassembly of AJC and reorganization of perijunctional F-actin following calcium repletion

**Figure 4:**
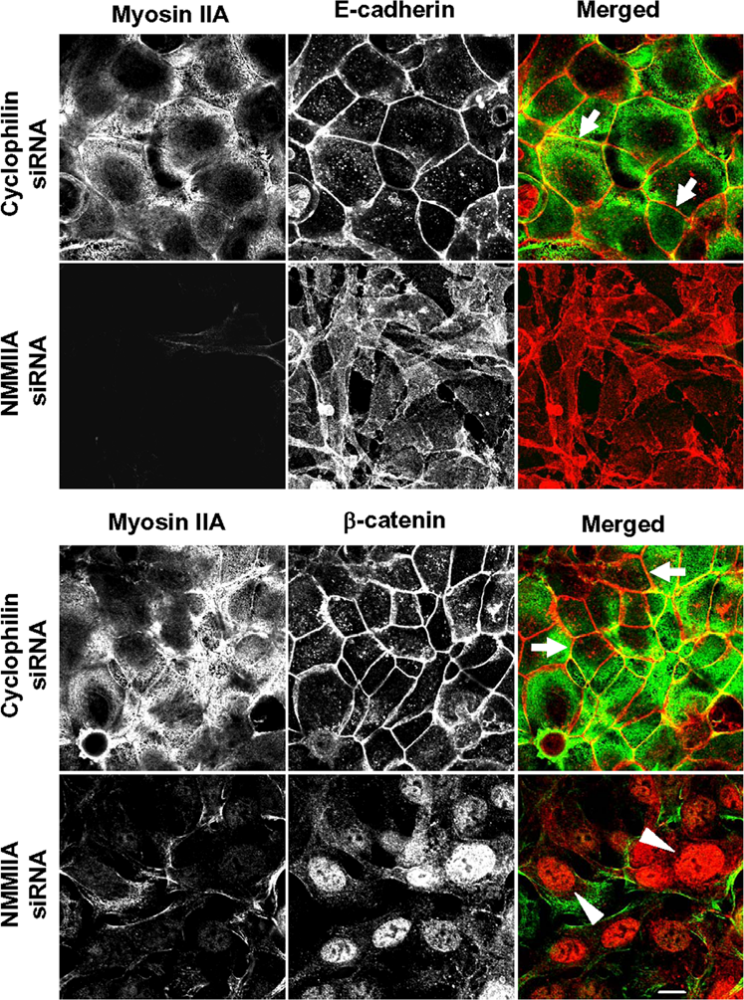
Downregulation of the NMMIIA expression impedes reformation of initial adherens-like junctions. SK-CO15 cells were transfected with either control (cyclophilin) or NMMII isoform specific siRNAs and on day 3 post-transfection were subjected to overnight calcium depletion in order to disrupt cell-cell adhesion. Reformation of initial adherens-like junctions was triggered by transferring cells for 1 h into the HCM. Control cells show rapid accumulation of E-cadherin and β-catenin (red) in areas of cell-cell contacts (arrows). In contrast, the majority of E-cadherin remains in the cytosol and β-catenin localizes in the nuclei in NMMIIA-depleted cells (arrowheads). Bar, 10 µm.

**Figure 5 pone-0000658-g005:**
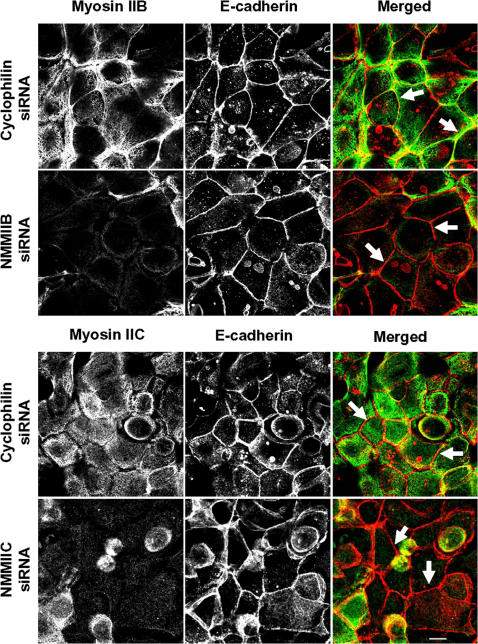
siRNA-mediated knock-down of either NMMIIB or NMMIIC has no effect on reformation of initial adherens-like junctions. Control, NMMIIB or NMMIIC siRNA-transfected SK-CO15 monolayers were subjected to overnight calcium depletion to disrupt cell-cell adhesion. Reformation of the initial adherens-like junctions was triggered by transferring cells for 1 h into the HCM. Similarly to control cells, NMMIIB and NMMIIC knock-down rapidly translocate E-cadherin (red) to areas of cell-cell contacts (arrows). Bar, 10 µm.

Several recent studies have demonstrated that siRNA-mediated downregulation of major junctional proteins such as E-cadherin and ZO-1 did not prevent the eventual formation of the AJC, but resulted in decreased rate of junctional assembly [51], [52]. These observations led us to examine whether downregulation of NMMII isoforms affected the dynamics of reassembly of epithelial AJs and TJs using a well established “calcium switch” model [53]. SK-CO15 cells were transfected with either control or NMMII isoform specific siRNAs and on day 3 post-transfection were transferred to a low-calcium medium (LCM; ∼5 µM of Ca^2+^) overnight in order to disrupt intercellular junctions. AJC reassembly was then triggered by switching from LCM to a high calcium medium (HCM; ∼1.8 mM of Ca^2+^). Since AJC reassembly in colonic epithelium occurs in two stages involving early formation of nascent AJ-like junctions followed by assembly of TJs [18], we investigated which stage is affected by myosin II knock-down. Overnight incubation in LCM resulted in SK-CO15 cell rounding and a loss of the majority of cell-cell contacts along with intracellular accumulation of AJ and TJ proteins (data not shown). Within the first hour of calcium reintroduction, the majority of cells in control monolayers acquired AJ-like junctions enriched in E-cadherin and β-catenin (Figure 4, arrows). Similar to control cell monolayers, NMMIIB or NMMIIC-depleted cells rapidly reassembled E-cadherin (Figure 5, arrows) and β-catenin (data not shown)-based AJ-like junctions. In stark contrast, formation of early AJ-like junctions was dramatically attenuated by NMMIIA knock-down. After 1 h of calcium repletion, there was very little accumulation of E-cadherin or β-catenin at the intercellular contacts in NMMIIA-depleted cells (Figure 4). Furthermore, E-cadherin was diffusely localized in the cytoplasm, and β-catenin predominantly localized in the nuclei (Figure 4, arrowheads).

**Figure 6 pone-0000658-g006:**
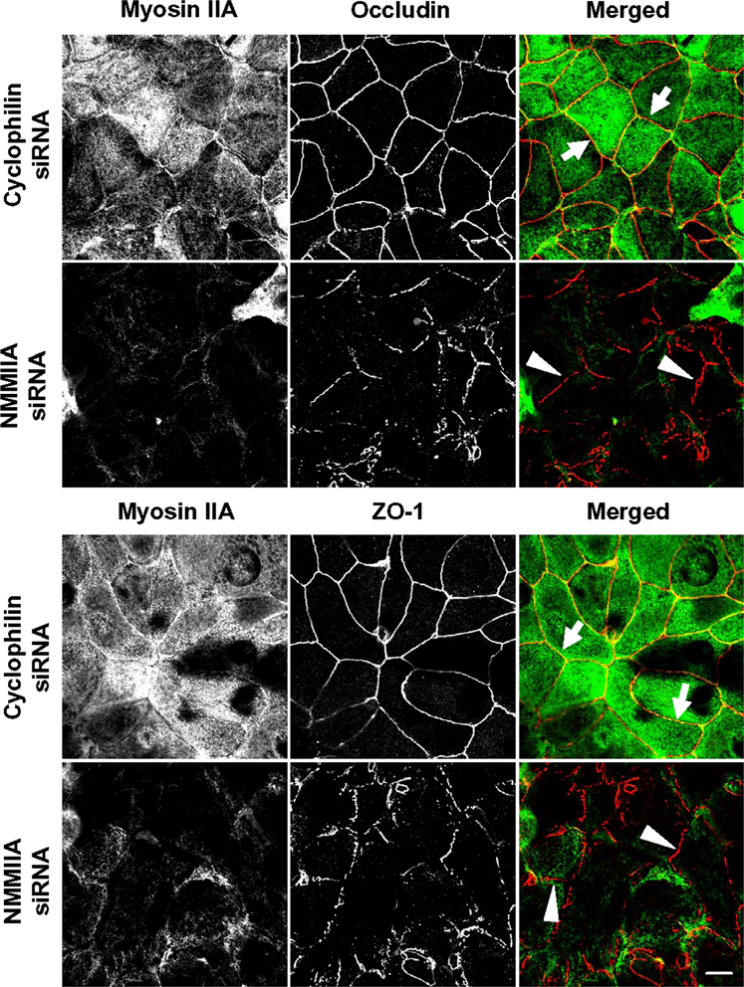
siRNA-mediated depletion of NMMIIA attenuates the development of tight junctions. SK-CO15 cells were transfected with either control (cyclophilin) or NMMII isoform specific siRNAs and on day 3 post-transfection were subjected to overnight calcium depletion in order to disrupt cell-cell adhesion. Reassembly of TJs in control and NMMIIA-deficient cells was investigated after 5 h of calcium repletion by monitoring the formation of characteristic ‘chicken wire’ labeling pattern of the TJ proteins occludin and ZO-1 (red). Control SK-CO15 cell monolayers show almost complete restoration of normal localization of occludin and ZO-1 at TJs (arrows). In contrast, occludin and ZO-1 labeling demonstrates abnormal discontinuous pattern at TJs in NMMIIA-deficient cells (arrowheads). Bar, 10 µm.

**Figure 7:**
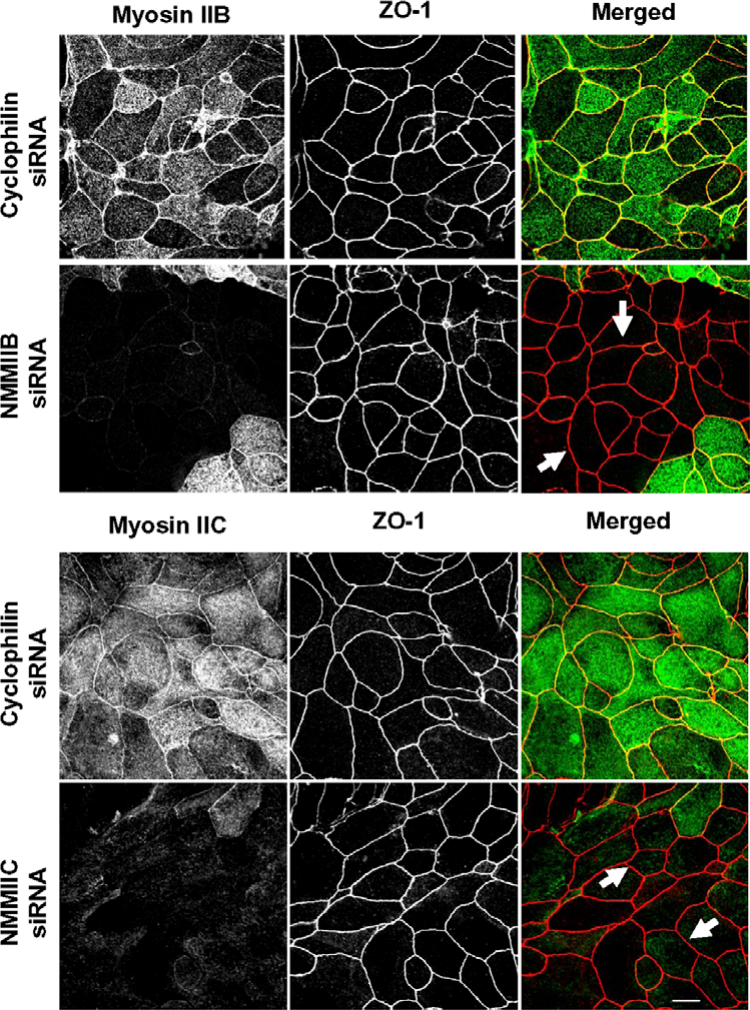
Downregulation of either NMMIIB or NMMIIC has no effect on reformation of epithelial TJs. Control, NMMIIB or NMMIIC siRNA-transfected SK-CO15 monolayers were subjected to overnight calcium depletion to disrupt cell-cell adhesion. Reformation of TJs was triggered by transferring cells for 5 h into the HCM. Similarly to control cells, NMMIIB and NMMIIC-deficient cell monolayers rapidly restore normal junctional labeling pattern for ZO-1 (arrows). Bar, 10 µm.

After 5–6 h of calcium repletion, control SK-CO15 cell monolayers had consistently reestablished normal TJs with characteristic ‘chicken wire’ staining of occludin and ZO-1 (Figure 6, arrows). In contrast, NMMIIA-deficient cells demonstrated discontinuous and disorganized labeling of occludin and ZO-1 at the areas of cell-cell contacts (Figure 6, arrowheads). Similar to control cell monolayers, NMMIIB and NMMIIC-deficient cells had normal junctional localization of ZO-1 (Figure 7, arrows) and occludin (data not shown) within 5 h following calcium repletion. Overall, these data demonstrate that expressional down-regulation of NMMIIA dramatically attenuates formation of both nascent AJ-like junctions and TJs, whereas depletion of two other NMMII isoforms does not affect the dynamics of AJC assembly.

**Figure 8:**
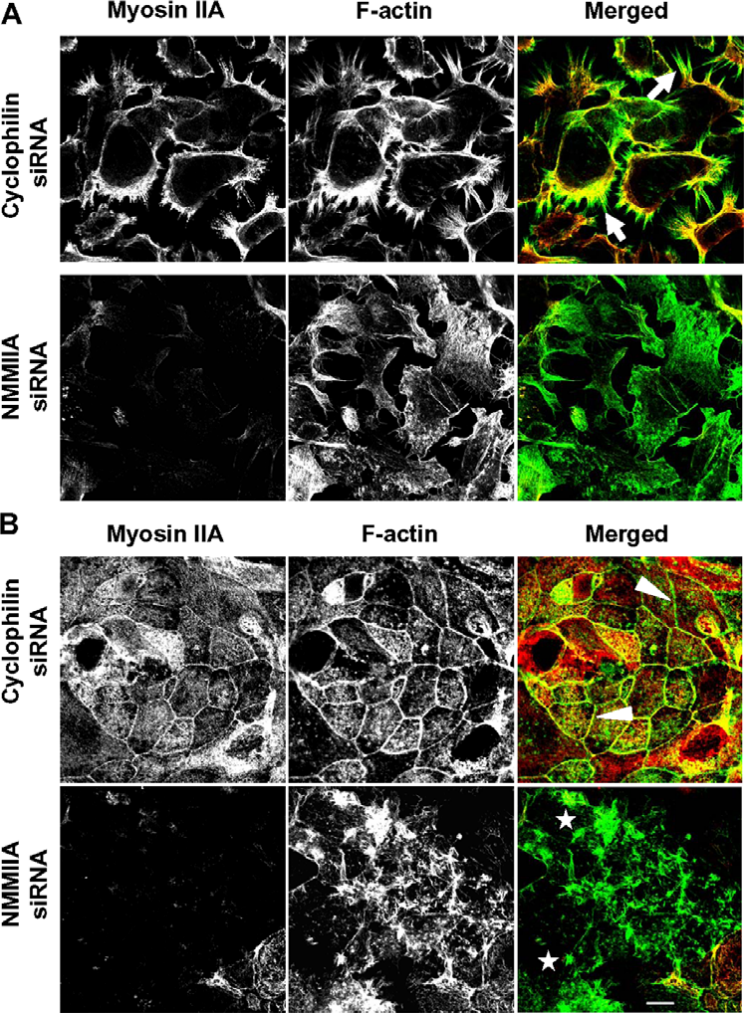
siRNA knock-down of NMMIIA causes disorganization of F-actin cytoskeleton. Calcium-depleted control and NMMIIA-deficient SK-CO15 cell monolayers were transferred into the HCM for 0.5 h (A) and 5 h (B) to trigger junctional reassembly. Organization of their actin filaments was visualized using fluorescently labeled phalloidin. At the early time of calcium repletion, prominent radial F-actin cables can be seen in lamellipodia of spreading/contacting control cells (A, arrows). At a later time, control cells show circumferential apical F-actin bundles (B, arrowheads). Neither structure is formed in NMMIIA-deficient cells which show diffuse (A) or abnormally aggregated (B, stars) F-actin. Bar, 10 µm.

Since the major function of myosin II involves translocation and bundling of F-actin, we suggested that NMMIIA might mediate assembly of the AJC by regulating structure and dynamics of perijunctional actin microfilaments. To test this suggestion, we analyzed reorganization of cortical F-actin in control and NMMIIA-deficient cell monolayers subjected to calcium switch. At early time points (0.5–1 h) of calcium repletion, spreading control SK-CO15 cells formed abundant lamellipodia with prominent radial actomyosin cables (Figure 8A, arrows). When the lamellipodia of two neighboring cells collided, such radial actomyosin cables accumulated AJ proteins, E-cadherin and β-catenin creating initial junctions (data not shown). At later time points (5–6 h) of calcium-repletion, radial actomyosin cables were replaced by apical circumferential bundles at the level of AJC (Figure 8B, arrowheads). In contrast, the architecture of cortical/perijunctional F-actin was markedly altered in NMMIIA-deficient cells. After 0.5 h of calcium repletion, there were few actomyosin cables and thin peripheral protrusions with diffuse/disorganized cortical F-actin (Figure 8A). Likewise, NMMIIA-depleted cells did not form circumferential perijunctional actin bundles at later time points of calcium repletion. In these cells, the apical F-actin appeared organized into abnormal aster-like aggregates (Figure 8B, stars). These results strongly suggest that attenuated assembly of AJ-like junctions and TJs in NMMIIA-depleted cells is likely to be a consequence of disorganization of cortical and perijunctional actin microfilaments.

### Downregulation of NMMIIA attenuated contraction-driven disassembly of the AJC during calcium depletion

**Figure 9:**
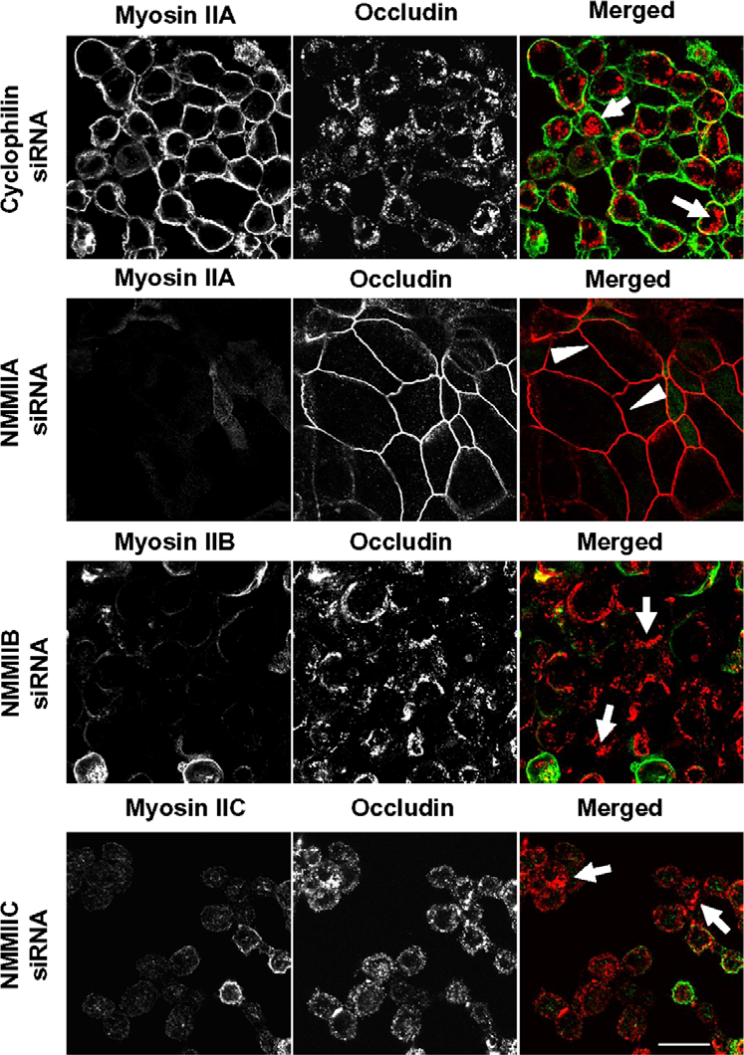
siRNA-mediated knock-down of NMMIIA selectively attenuates disassembly of the AJC in calcium-depleted cells. Control, NMMIIA, NMMIIB, and NMMIIC-deficient SK-CO15 monolayers were incubated for 1 h in the LCM-EGTA, and the integrity of their AJC was analyzed by immunolabeling for occludin (red). Calcium depletion causes rapid disruption of the AJC and accumulation of occludin in cytosolic ring-like structures in control, NMMIIB and NMMIIC-deficient cells (arrows). In contrast, the majority of occludin-labeled TJs remained intact in cells monolayers subjected to NMMIIA knock-down (arrowheads). Bar, 20 µm.

**Figure 10:**
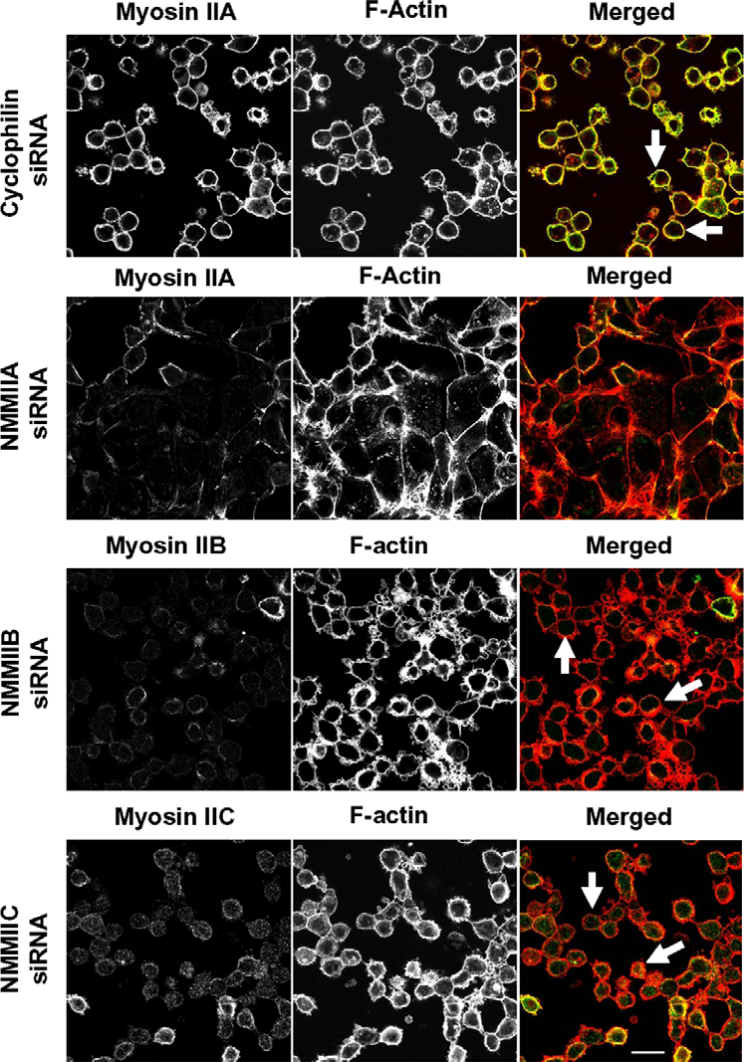
siRNA-mediated knock-down of NMMIIA inhibits cell contractility triggered by calcium-depletion. Control, NMMIIA, NMMIIB, and NMMIIC-deficient SK-CO15 monolayers were subjected to 1 h calcium depletion, and their overall cell shape was analyzed by F-actin labeling. Calcium depletion causes rapid rounding of control, NMMIIB, and NMMIIC-deficient cells (arrows). In contrast, cell rounding was significantly attenuated in cells monolayers subjected to NMMIIA knock-down. Bar, 20 µm.

Previous pharmacological inhibition studies have demonstrated that myosin II-driven contraction plays a critical role in disassembly of the AJC triggered by different extracellular stimuli [17], [25], [54]. Therefore, we next investigated the role of myosin II isoforms in regulating disruption of epithelial apical junctions. SK-CO15 cells were transfected on coverslips with either control or NMMII isoforms-specific siRNAs, and 4 days later were subjected to 1 h calcium depletion in LCM-EGTA to trigger junctional disassembly. Calcium depletion of control cells caused rapid translocation of occludin from cell-cell junctions into intracellular dot-like aggregates (Figure 9, arrows), thus indicating breakdown and internalization of the AJC. Similar changes in occludin labeling were observed in cells with NMMIIB and NMMIIC knock-downs (Figure 9, arrows). In contrast, the majority of occludin remained in cell-cell junctions in calcium depleted NMMIIA deficient cell monolayers (Figure 9, arrowheads) suggesting attenuation in AJC disassembly. To test whether altered cell contractility mediated such effects, we analyzed cells for changes in shape using F-actin labeling. After 1 h of calcium depletion, the majority of cells in control, NMMIIB and NMMIIC-deficient SK-CO15 monolayers appeared uniformly rounded and detached from many neighboring cells (Figure 10, arrows) indicating cell contraction. In contrast, NMMIIA-deficient cell did not round up, and remained spread over the substratum (Figure 10). These data suggest that NMMIIA plays a unique role in contraction-driven disassembly of epithelial AJC during calcium depletion.

## Discussion

In this study, we report two major findings. We provide the first direct non-pharmacological evidence that myosin II controls reorganization (assembly and disassembly) of the AJC in mammalian epithelia. In addition, we report that NMMIIA plays a unique role in the regulation of epithelial adherens and tight junctions.

### All three NMMII isoforms are co-expressed in human intestinal epithelium and colocalize at the AJC

We observed that well differentiated human intestinal epithelial cells such as SK-CO15 and Caco-2 cells express all three NMMII isoforms at both the mRNA and protein level (Figure 1). NMMIIA, NMMIIB and NMMIIC were enriched at the apical circumferential F-actin belt and colocalized with the AJC in cultured cell monolayers (Figure 2A). Despite similar localization, NMMIIA, NMMIIB and NMMIIC do not form heterodimers in intestinal epithelial cells (Figure 2B), which is a critical prerequisite for functional diversity of the NMMII heavy chains. Furthermore, our data suggest more labile association of NMMIIA with F-actin compared to the other isoforms (Figure 2C), which may be due to reported differences in kinetic mechanisms for NMMIIA and NMMIIB. In particular, NMMIIA is a low-duty-ratio motor, which is not attached to F-actin during most of the kinetic cycle [55]. In contrast, NMMIIB is an intermediate-duty-ratio motor, spending a higher proportion of its kinetic cycle firmly bound to actin [56], [57]. These different biochemical properties of NMMII isoforms may determine their functional peculiarities, with NMMIIB being suitable to maintain static tension and NMMIIA being adapted to rapidly reorganize actin microfilaments.

### NMMIIA regulates the development of epithelial barrier but is dispensable for the maintenance of normal AJC structure

We obtained direct evidence of non-redundant functions of NMMIIA in SK-CO15 cells by isoform-specific siRNA-mediated knock-down. Depletion of NMMIIA but not NMMIIB and NMMIIC resulted in markedly altered cell shape (Figure 3B) and was characterized by enhanced paracellular permeability (Figure 3C). Interestingly, confluent monolayers of NMMIIA-depleted cells possessed morphologically-normal AJs and TJs (Figures S1 and S2) and did not show changes in the expression of AJ/TJ proteins (Figure S3) This data seemingly contradicts to two recent studies which reported decreased accumulation of E-cadherin and β-catenin at intercellular contacts of mouse embryonic stem cells and COS-7 embryonic kidney epithelial cells after siRNA knock-down of NMMIIA [44] and NMMIIB [26] respectively. However, mouse embryonic stem cells do not express NMMIIC [44], and targeted elimination of NMMIIA would result in NMMIIA/NMMIIC-deficient cells. Likewise, COS-7 cells lack the NMMIIA expression [38] and elimination of NMMIIB would lead to dual NMMIIA/NMMIIB deficiency. Thus, it is not surprising that such a lack of two myosin II-isoforms would result in more severe defects of apical junctions comparing to selective knock-down of NMMIIA. Depletion of individual myosin II isoforms revealed that NMMIIB and NMMIIC are not involved in maintenance of normal structure and barrier function of the AJC, whereas NMMIIA is essential for maintenance of paracellular barrier but not structural integrity of epithelial AJs and TJs (Figures 3, S1 and S2).

### NMMIIA plays a critical role in the formation and disassembly of the AJC

Our results highlight a unique role of NMMIIA in the dynamic reorganization (assembly and disassembly) of epithelial AJs and TJs (Figures 4–7). It is noteworthy that previous pharmacological inhibition studies produced conflicting results on the involvement of myosin II in the assembly of AJs. For example, inhibition of myosin II with blebbistatin was reported to have no effect on formation of AJ-like junctions in T84 [18] and MTD-1A [26] epithelial cells, but reportedly decreased junctional accumulation of E-cadherin in keratinocytes [21] and MCF-7 cells [27]. However, blebbistatin is a relatively low affinity myosin II inhibitor [58] and cells treated with the drug at concentration of 50–100 µM retain a significant level of myosin II activity [39]. Thus, differences in the extent of myosin II inhibition by blebbistatin may explain these apparent discrepancies. The siRNA knock-down results in this study clearly implicate myosin II in the formation of AJs in intestinal epithelium and highlight a unique role for NMMIIA in this process (Figures 4 and 5). Furthermore, our results demonstrate a critical role of NMMIIA in the assembly of TJs (Figures 6 and 7) thus reinforcing previous pharmacological data [18], [21], [26] and revealing the molecular identity of the myosin II motor that regulates sealing of the epithelial barrier.

We also determined that NMMIIA not only controls the assembly of AJs and TJs, but regulates disassembly of epithelial AJC. It has been suggested that the mechanisms governing disruption of AJs and TJs caused by calcium depletion [17], or cell exposure to proinflammatory cytokines [24], [25] and growth factors [54] involve myosin II-dependent contraction of perijunctional F-actin belt. Our results suggest that, in calcium-depleted cells, such contraction-driven AJC disassembly requires the activity of NMMIIA but not other myosin II isoforms (Figures 9 and 10). This data is consistent with a recent report that NMMIIA but not NMMIIB mediates thrombin-induced rounding of fibroblast-like MDA-231 cells [41] and suggest a common role for NMMIIA in the regulation of rapid contractile responses to various extracellular stimuli.

### NMMIIA controls formation of cortical and perijunctional F-actin bundles during assembly of the AJC

The observed effects of NMMIIA depletion on assembly of the AJC and cell polarization are likely to be a consequence of dramatic changes in the actin cytoskeleton. Major types of F-actin-rich structures mediating the formation of epithelial AJC include radial cables (Figure 8), that are essential for the assembly of initial E-cadherin-based junctions [16], [18], [20], [28] and circumferential perijunctional bundles (Fig 6) that regulate maturation of the AJC and apico-basal cell polarization [18], [21]. Formation of radial cables and circumferential bundles was disrupted in NMMIIA-depleted cells resulting in diffuse F-actin structures or abnormal apical F-actin aggregates at different stages of AJC reassembly (Figure 8).

A current model suggests a limited role of myosin II in the biogenesis of intercellular junctions [19]–[21], [59]. Thus, assembly of radial F-actin cables that support initial E-cadherin-based cell-cell contacts is thought to be myosin II-independent and driven purely by actin polymerization [60], [61]. In this model, myosin II-mediated contraction creates lateral tension that expands the initial cell-cell adhesions [59]. However, our results suggest that NMMIIA may not only serve as a “zipper” for E-cadherin-based junctions, but controls the formation of radial F-actin cables that initiate AJC assembly. This observation is supported by recent data obtained in migrating epithelial cells where NMMIIA knock-down was shown to disperse stress fibers [39], [41], thus resembling disappearance of radial F-actin cables observed in the present study.

In summary, the present study highlights a critical role for NMMIIA in regulation of cell-cell adhesion and paracellular permeability in human epithelia. There appears to be limited redundancy in these NMMIIA functions as defects in junctional dynamics caused by NMMIIA depletion are not compensated for by the remaining myosin II isoforms. Further studies are needed to better understand the cellular mechanisms that determine unique functions of NMMIIA and other myosin II isoforms in human epithelia.

## Materials and Methods

### Antibodies and Other Reagents

The following primary polyclonal (pAb) and monoclonal (mAb) antibodies were used to detect AJC proteins and myosin II isoforms: anti NMMIIA and NMMIIB pAbs (Covance, Berkley, CA); anti-occludin, ZO-1, claudin-1, cingulin, and JAM-A pAbs (Zymed Laboratories, San Francisco, CA); anti-ZO-1, occludin, E-cadherin, β-catenin mAbs (Zymed). Anti-NMMIIC pAb was previously described [40]. Alexa-488 or Alexa-568 dyes conjugated donkey anti-rabbit and goat anti-mouse secondary antibodies G-and Alexa-labelled phalloidin, were obtained from Molecular Probes (Eugene, OR); horseradish peroxidase-conjugated goat anti-rabbit and anti-mouse secondary antibodies were obtained from Jackson Immunoresearch Laboratories (West Grove, PA). All Other reagents were of the highest analytical grade and were obtained from Sigma.

### Cell Culture

SK-CO-15, a transformed human colonic epithelial cell line, was a gift from Dr. Enrique Rodriguez-Boulan, Weill Medical College of Cornell University, New York. SK-CO-15, and Caco-2 human colonic epithelial cells (American Type Culture Collection, Manassas, VA) were grown in Dulbecco's modified Eagle's medium supplemented with 10% fetal bovine serum, 2 mM l-glutamine, 15 mM HEPES, 1% nonessential amino acids, 40 µg/ml penicillin and 100 µg/ml streptomycin, pH 7.4. This medium is designated as HCM. T84 human colonic epithelial cells (American Type Culture Collection) were cultured as previously described [17], [18], [45]. For immunolabeling experiments, epithelial cells were grown on either collagen-coated, permeable polycarbonate filters 0.4 µm pore size (Costar, Cambridge, MA) or on collagen-coated cover slips. For biochemical experiments, the cells were cultured on 6-well plastic plates.

### Calcium Switch Model

To study formation of epithelial TJs and AJs, confluent SK-CO15 monolayers were first depolarized by overnight incubation in LCM (calcium-free Eagle's minimum essential medium for suspension culture (Sigma) supplemented with 10 mM HEPES, 14 mM NaHCO_3_, 40 µg/ml penicillin, 100 µg/ml streptomycin, 5 µM CaCl_2_ and 10% dialyzed fetal bovine serum, pH 7.4). To induce reassembly of the AJC, the cells were returned to the HCM for indicated times at 37°C. To induce a rapid disassembly of the AJC, SK-CO15 cells were incubated for 1 h in the LCM without CaCl_2_ supplemented with 2 mM EGTA (designated as LCM-EGTA).

### Immunofluorescence Labeling and Image Analysis

Cell monolayers were fixed/permeabilized in 100% methanol (−20°C for 20 min), blocked in HEPES-buffered Hanks balanced salt solution (HBSS^+^) containing 1% bovine serum albumin (blocking buffer) for 60 min at room temperature and incubated for another 60 min with primary antibodies diluted in blocking buffer. Cells were then washed, incubated for 60 min with Alexa dye-conjugated secondary antibodies, rinsed with blocking buffer and mounted on slides with ProLong Antifade medium (Molecular Probes). For fluorescent double-labeling of myosin II isoforms with F-actin, monolayers were fixed in 100% ethanol (−20°C for 20 min) and sequentially stained with primary anti-myosin II heavy chain and Alexa dye-conjugated secondary antibodies, whereas F-actin was labeled with Alexa-conjugated phalloidin. Stained cell monolayers and tissue sections were examined using a Zeiss LSM510 laser scanning confocal microscope (Zeiss Microimaging Inc., Thornwood, NY) coupled to a Zeiss 100M axiovert and 63× or 100× Pan-Apochromat oil lenses. The fluorescent dyes were imaged sequentially in frame-interlace mode to eliminate cross talk between channels. Images were processed using Zeiss LSM5 image browser software and Adobe Photoshop. Images shown are representative of at least 3 experiments, with multiple images taken per slide.

### Immunoblotting

Cells were homogenized in a RIPA lysis buffer (20 mM Tris, 50 mM NaCl, 2 mM EDTA, 2 mM EGTA, 1% sodium deoxycholate, 1% TX-100, and 0.1% SDS, pH 7.4), containing a proteinase inhibitor cocktail (1∶100, Sigma) and phosphatase inhibitor cocktails 1 and 2 (both at 1∶200, Sigma). Lysates were then cleared by centrifugation (20 min at 14,000 × g), diluted with 2× SDS sample buffer and boiled. Polyacrylamide gel electrophoresis and Western blotting were conducted by standard protocols with 10–20 µg protein per lane. Results shown are representative immunoblots of three independent experiments. Protein expression was quantified by densitometric analysis of Western blot images on UN-SCAN-IT digitizing software (Silk Scientific, Orem, UT).

### Immunoprecipitation

Confluent monolayers of SK-CO15 and Caco-2 cells were homogenized in an immunoprecipitation buffer (50 mM PIPES, 50 mM HEPES, 1 mM EDTA, 2 mM MgSO_4_, 1% TX-100, and 0.5% Igepal, pH 7.0), supplemented with proteinase and phosphatase inhibitors. Cell debris-free supernatants (500 µl) were precleared with Protein A-coupled Sepharose beads (Amersham Biosciences, Buckinghamshire, UK) for 60 min at 4°C followed by overnight incubation at 4°C with 5 µg of either anti-NMMIIA, NMMIIB, NMMIIC pAbs or control rabbit IgG (Jackson Laboratories). Immunocomplexes were recovered by incubation with Protein A-Sepharose beads for 3 h at 4°C with constant rotation. The washed beads were boiled for 5 min in 80 µl of 2× SDS sample buffer and equal volumes of supernatants (20 µl) were analyzed by electrophoresis and Western blotting as described above.

### Triton X-100 Fractionation

Determination of the relative amounts of F-actin- bound and unbound myosin II was performed by TX-100 fractionation as previously described [62]. Briefly, SK-CO15 or Caco-2 monolayers were washed with HBSS^+^ and extracted for 15 min at 4°C with HBSS^+^ containing 1% TX-100, proteinase inhibitors, and 1 µg/ml phalloidin to prevent actin filament disassembly. The TX-100-soluble fraction was mixed with an equal volume of the 2×-SDS sample buffer and boiled. The TX-100-insoluble fraction was collected by scraping pre-extracted, filter-bound cells in two volumes of 1×-SDS sample buffer with subsequent homogenizing and boiling. The amount of NMMII in each fraction was determined by gel electrophoresis and Western blotting.

### RT-PCR

Total mRNA was isolated from SK-CO15, Caco-2 and T84 cells using RNAeasy Mini Kit (Qiagen, Valencia, CA) according to manufacturer's protocol. RT PCR was carried out using a Super Script III One Step RT PCR system (Invitrogen). The primers sequences are presented in Table 1. Specificity of amplification was verified buy running agarose electrophoresis of each amplicon and obtaining a single band of expected size (358 bp, 323 bp and 220 bp for NMMIIA, NMMIIB, and NMMIIC respectively). All obtained PCR products were independently identified by sequencing.

### RNA interference

siRNA-mediated knock-down of NMMIIA, NMMIIB and NMMIIC was carried out using either isoform-specific siRNA SmartPools or individual siRNA duplexes (Dharmacon, Lafayette, CO; see Table 1 for sequences). Cyclophilin B siRNA SmartPool or individual cyclophilin B siRNA (5′-ucaccguagaugcucuuucuu) were used as controls. SK-CO15 cells were transfected using the DharmaFect 1 transfection reagent (Dharmacon) in Opti-MEM I medium (Invitrogen) according to manufacturer's protocol with a final siRNA concentration of 100 nM. Cells were used in experiments 72–80 h post-transfection.

### Transepithelial Electrical Resistance Measurement

Effect of calcium depletion on transepithelial electrical resistance was measured using an EVOMX voltohmmeter (World Precision Instruments, Sarasota, FL). The resistance of cell-free collagen-coated filters was subtracted from each experimental point.

### Statistics

Numerical values from individual experiments were pooled and expressed as mean±standard error of the mean (S.E.) throughout. Obtained numbers were compared by a single-tailed Student's *t* test, with statistical significance assumed at p<0.05.

## Supporting Information

Figure S1 siRNA-mediated knock-down of NMMII isoforms does not affect the morphology of mature epithelial AJs. SK-CO15 cells were transfected with either control, NMMIIA, NMMIIB, or NMMIIC siRNAs and on day 4 post-transfection were double-immunolabeled for myosin II heavy chains (green) and E-cadherin (red). Control cells and cells with the myosin II isoforms knock-down show predominant localization of E-cadherin at areas of cell-cell contact which is characteristic of normal AJs (arrows).
(2.68 MB TIF)

Figure S2 siRNA-mediated knock-down of individual NMMII isoforms does not affect the morphology of mature epithelial TJs. SK-CO15 cells were transfected with either control, NMMIIA, NMMIIB, or NMMIIC siRNAs and on day 4 post-transfection were double-immunolabeled for myosin II heavy chains (green) and occludin (red). Similar to control monolayers, cells with NMMIIA, NMMIIB, and NMMIIC knock-down show a ‘chicken wire’ labeling pattern for occludin (arrows) indicative of normal TJs.
(2.85 MB TIF)

Figure S3 siRNA-mediated down-regulation of NMMII isoforms has no effect on the expression of major AJ/TJ proteins. SK-CO15 cells were transfected with either control, NMMIIA, NMMIIB, or NMMIIC siRNAs and 4 days later were analyzed for expression of different AJ and TJ proteins by Western blotting. Note that expression of junctional proteins was not affected by down-regulation of individual NMMII isoforms.
(0.34 MB TIF)

## References

[1] Anderson JM, Van Itallie CM, Fanning AS (2004). Setting up a selective barrier at the apical junction complex.. Curr Opin Cell Biol.

[2] Shin K, Fogg VC, Margolis B (2006). Tight junctions and cell polarity.. Annu Rev Cell Dev Biol.

[3] Tsukita S, Furuse M, Itoh M (2001). Multifunctional strands in tight junctions.. Nat Rev Mol Cell Biol.

[4] Aijaz S, Balda MS, Matter K (2006). Tight junctions: molecular architecture and function.. Int Rev Cytol.

[5] Blaschuk OW, Rowlands TM (2002). Plasma membrane components of adherens junctions.. Mol Membr Biol.

[6] Ogita H, Takai Y (2006). Nectins and nectin-like molecules: roles in cell adhesion, polarization, movement, and proliferation.. IUBMB Life.

[7] Wheelock MJ, Johnson KR (2003). Cadherins as modulators of cellular phenotype.. Annu Rev Cell Dev Biol.

[8] Yap AS, Brieher WM, Gumbiner BM (1997). Molecular and functional analysis of cadherin-based adherens junctions.. Annu Rev Cell Dev Biol.

[9] Gonzalez-Mariscal L, Betanzos A, Nava P, Jaramillo BE (2003). Tight junction proteins.. Prog Biophys Mol Biol.

[10] Goodwin M, Yap AS (2004). Classical cadherin adhesion molecules: coordinating cell adhesion, signaling and the cytoskeleton.. J Mol Histol.

[11] Weis WI, Nelson WJ (2006). Re-solving the cadherin-catenin-actin conundrum.. J Biol Chem.

[12] Mooseker MS (1985). Organization, chemistry, and assembly of the cytoskeletal apparatus of the intestinal brush border.. Annu Rev Cell Biol.

[13] Madara JL (1998). Regulation of the movement of solutes across tight junctions.. Annu Rev Physiol.

[14] Stevenson BR, Begg DA (1994). Concentration-dependent effects of cytochalasin D on tight junctions and actin filaments in MDCK epithelial cells.. J Cell Sci.

[15] Shen L, Turner JR (2005). Actin depolymerization disrupts tight junctions via caveolae-mediated endocytosis.. Mol Biol Cell.

[16] Adams CL, Chen YT, Smith SJ, Nelson WJ (1998). Mechanisms of epithelial cell-cell adhesion and cell compaction revealed by high-resolution tracking of E-cadherin-green fluorescent protein.. J Cell Biol.

[17] Ivanov AI, McCall IC, Parkos CA, Nusrat A (2004). Role for actin filament turnover and a myosin II motor in cytoskeleton-driven disassembly of the epithelial apical junctional complex.. Mol Biol Cell.

[18] Ivanov AI, Hunt D, Utech M, Nusrat A, Parkos CA (2005). Differential roles for actin polymerization and a myosin II motor in assembly of the epithelial apical junctional complex.. Mol Biol Cell.

[19] Vaezi A, Bauer C, Vasioukhin V, Fuchs E (2002). Actin cable dynamics and Rho/Rock orchestrate a polarized cytoskeletal architecture in the early steps of assembling a stratified epithelium.. Dev Cell.

[20] Vasioukhin V, Bauer C, Yin M, Fuchs E (2000). Directed actin polymerization is the driving force for epithelial cell-cell adhesion.. Cell.

[21] Zhang J, Betson M, Erasmus J, Zeikos K, Bailly M (2005). Actin at cell-cell junctions is composed of two dynamic and functional populations.. J Cell Sci.

[22] Gandhi S, Lorimer DD, de Lanerolle P (1997). Expression of a mutant myosin light chain that cannot be phosphorylated increases paracellular permeability.. Am J Physiol.

[23] Hecht G, Pestic L, Nikcevic G, Koutsouris A, Tripuraneni J (1996). Expression of the catalytic domain of myosin light chain kinase increases paracellular permeability.. Am J Physiol.

[24] Turner JR (2006). Molecular basis of epithelial barrier regulation: from basic mechanisms to clinical application.. Am J Pathol.

[25] Utech M, Ivanov AI, Samarin SN, Bruewer M, Turner JR (2005). Mechanism of IFN-γ-induced endocytosis of tight junction proteins: myosin II-dependent vacuolarization of the apical plasma membrane.. Mol Biol Cell.

[26] Miyake Y, Inoue N, Nishimura K, Kinoshita N, Hosoya H (2006). Actomyosin tension is required for correct recruitment of adherens junction components and zonula occludens formation.. Exp Cell Res.

[27] Shewan AM, Maddugoda M, Kraemer A, Stehbens SJ, Verma S (2005). Myosin 2 is a key Rho kinase target necessary for the local concentration of E-cadherin at cell-cell contacts.. Mol Biol Cell.

[28] Gloushankova NA, Krendel MF, Alieva NO, Bonder EM, Feder HH (1998). Dynamics of contacts between lamellae of fibroblasts: essential role of the actin cytoskeleton.. Proc Natl Acad Sci U S A.

[29] Ma TY, Tran D, Hoa N, Nguyen D, Merryfield M (2000). Mechanism of extracellular calcium regulation of intestinal epithelial tight junction permeability: role of cytoskeletal involvement.. Microsc Res Tech.

[30] Ostap EM (2002). 2,3-Butanedione monoxime (BDM) as a myosin inhibitor.. J Muscle Res Cell Motil.

[31] Yarrow JC, Lechler T, Li R, Mitchison TJ (2003). Rapid de-localization of actin leading edge components with BDM treatment.. BMC Cell Biol.

[32] Shu S, Liu X, Korn ED (2005). Blebbistatin and blebbistatin-inactivated myosin II inhibit myosin II-independent processes in *Dictyostelium*.. Proc Natl Acad Sci U S A.

[33] Lecuit T (2005). Adhesion remodeling underlying tissue morphogenesis.. Trends Cell Biol.

[34] De La Cruz EM, Ostap EM (2004). Relating biochemistry and function in the myosin superfamily.. Curr Opin Cell Biol.

[35] Krendel M, Mooseker MS (2005). Myosins: tails (and heads) of functional diversity.. Physiology (Bethesda).

[36] Golomb E, Ma X, Jana SS, Preston YA, Kawamoto S (2004). Identification and characterization of nonmuscle myosin II-C, a new member of the myosin II family.. J Biol Chem.

[37] Phillips CL, Yamakawa K, Adelstein RS (1995). Cloning of the cDNA encoding human nonmuscle myosin heavy chain-B and analysis of human tissues with isoform-specific antibodies.. J Muscle Res Cell Motil.

[38] Bao J, Jana SS, Adelstein RS (2005). Vertebrate nonmuscle myosin II isoforms rescue small interfering RNA-induced defects in COS-7 cell cytokinesis.. J Biol Chem.

[39] Cai Y, Biais N, Giannone G, Tanase M, Jiang G, Hofman JM (2006). Nonmuscle myosin IIA-dependent force inhibits cell spreading and drives F-actin flow.. Biophys J.

[40] Jana SS, Kawamoto S, Adelstein RS (2006). A specific isoform of nonmuscle myosin II-C is required for cytokinesis in a tumor cell line.. J Biol Chem.

[41] Sandquist JC, Swenson KI, Demali KA, Burridge K, Means AR (2006). Rho kinase differentially regulates phosphorylation of nonmuscle myosin II isoforms A and B during cell rounding and migration.. J Biol Chem.

[42] Togo T, Steinhardt RA (2004). Nonmuscle myosin IIA and IIB have distinct functions in the exocytosis-dependent process of cell membrane repair.. Mol Biol Cell.

[43] Wei Q, Adelstein RS (2000). Conditional expression of a truncated fragment of nonmuscle myosin II-A alters cell shape but not cytokinesis in HeLa cells.. Mol Biol Cell.

[44] Conti MA, Even-Ram S, Liu C, Yamada KM, Adelstein RS (2004). Defects in cell adhesion and the visceral endoderm following ablation of nonmuscle myosin heavy chain II-A in mice.. J Biol Chem.

[45] Ivanov AI, McCall IC, Babbin B, Samarin SN, Nusrat A (2006). Microtubules regulate disassembly of epithelial apical junctions.. BMC Cell Biol.

[46] Le Bivic A, Real FX, Rodriguez-Boulan E (1989). Vectorial targeting of apical and basolateral plasma membrane proteins in a human adenocarcinoma epithelial cell line.. Proc Natl Acad Sci U S A.

[47] Mandell KJ, Babbin BA, Nusrat A, Parkos CA (2005). Junctional adhesion molecule 1 regulates epithelial cell morphology through effects on β1 integrins and Rap1 activity.. J Biol Chem.

[48] Litten RZ, Martin BJ, Low RB, Alpert NR (1982). Altered myosin isozyme patterns from pressure-overloaded and thyrotoxic hypertrophied rabbit hearts.. Circ Res.

[49] Hossain MM, Crish JF, Eckert RL, Lin JJ, Jin JP (2005). h2-Calponin is regulated by mechanical tension and modifies the function of actin cytoskeleton.. J Biol Chem.

[50] Qin Y, Capaldo C, Gumbiner BM, Macara IG (2005). The mammalian Scribble polarity protein regulates epithelial cell adhesion and migration through E-cadherin.. J Cell Biol.

[51] Capaldo CT, Macara IG (2007). Depletion of E-cadherin disrupts establishment but not maintenance of cell junctions in Madin-Darby canine kidney epithelial cells.. Mol Biol Cell.

[52] McNeil E, Capaldo CT, Macara IG (2006). Zonula occludens-1 function in the assembly of tight junctions in Madin-Darby canine kidney epithelial cells.. Mol Biol Cell.

[53] Gumbiner B, Stevenson B, Grimaldi A (1988). The role of the cell adhesion molecule uvomorulin in the formation and maintenance of the epithelial junctional complex.. J Cell Biol.

[54] de Rooij J, Kerstens A, Danuser G, Schwartz MA, Waterman-Storer CM (2005). Integrin-dependent actomyosin contraction regulates epithelial cell scattering.. J Cell Biol.

[55] Kovacs M, Wang F, Hu A, Zhang Y, Sellers JR (2003). Functional divergence of human cytoplasmic myosin II: kinetic characterization of the non-muscle IIA isoform.. J Biol Chem.

[56] Rosenfeld SS, Xing J, Chen LQ, Sweeney HL (2003). Myosin IIB is unconventionally conventional.. J Biol Chem.

[57] Wang F, Kovacs M, Hu A, Limouze J, Harvey EV (2003). Kinetic mechanism of non-muscle myosin IIB: functional adaptations for tension generation and maintenance.. J Biol Chem.

[58] Kovacs M, Toth J, Hetenyi C, Malnasi-Csizmadia A, Sellers JR (2004). Mechanism of blebbistatin inhibition of myosin II.. J Biol Chem.

[59] Krendel MF, Bonder EM (1999). Analysis of actin filament bundle dynamics during contact formation in live epithelial cells.. Cell Motil Cytoskeleton.

[60] Bershadsky A (2004). Magic touch: how does cell-cell adhesion trigger actin assembly?. Trends Cell Biol.

[61] Mege RM, Gavard J, Lambert M (2006). Regulation of cell-cell junctions by the cytoskeleton.. Curr Opin Cell Biol.

[62] Cramer LP, Briggs LJ, Dawe HR (2002). Use of fluorescently labelled deoxyribonuclease I to spatially measure G-actin levels in migrating and non-migrating cells.. Cell Motil Cytoskeleton.

