# Supplementary figures and images for: A Unique Role for Nonmuscle Myosin Heavy Chain IIA in Regulation of Epithelial Apical Junctions
Source: PLoS One. 2007 Aug 1;2(8):e658. doi: 10.1371/journal.pone.0000658 (PMC1920554; doi:10.1371/journal.pone.0000658)

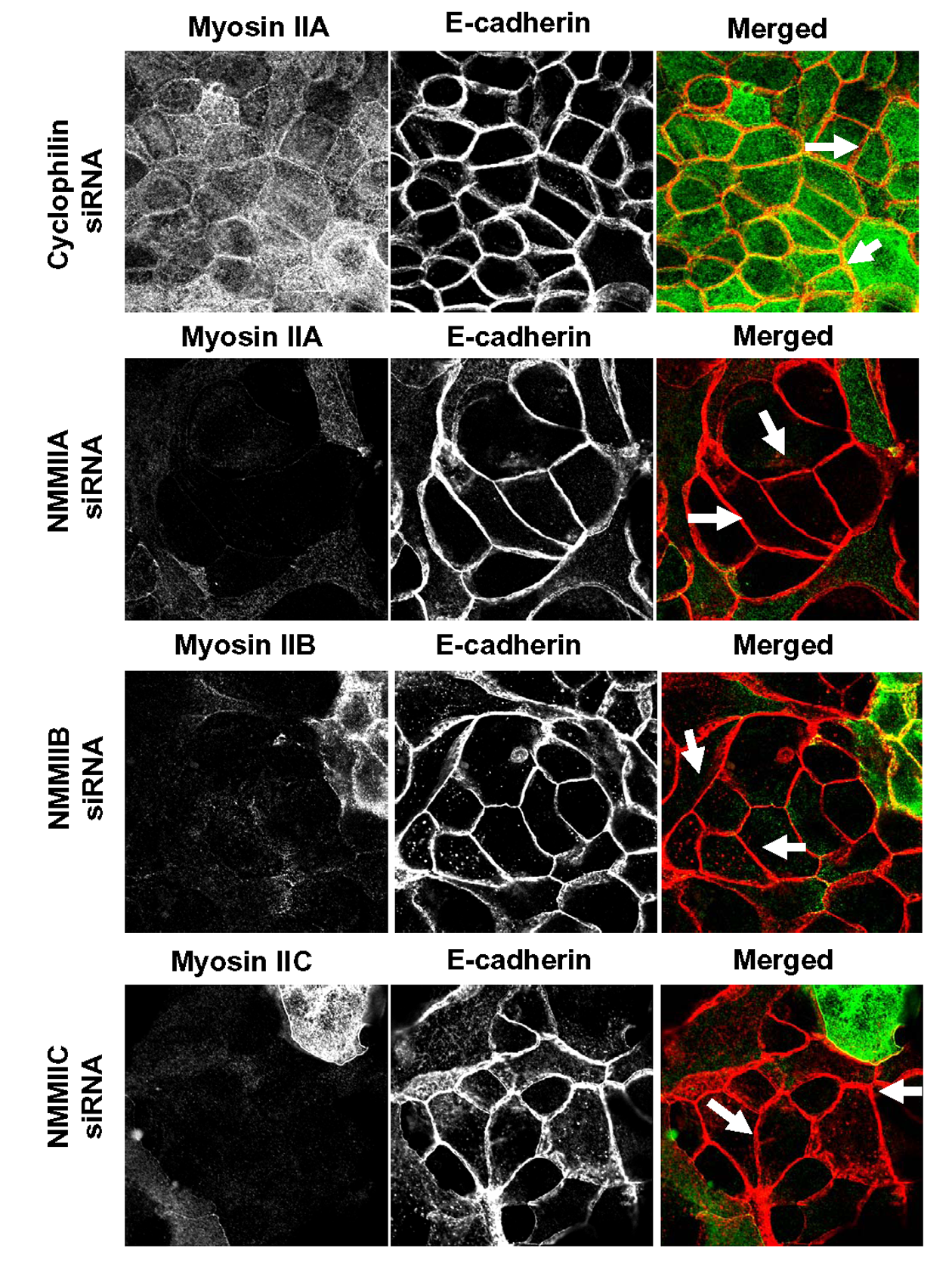

Supplement: Figure S1 — siRNA-mediated knock-down of NMMII isoforms does not affect the morphology of mature epithelial AJs. SK-CO15 cells were transfected with either control, NMMIIA, NMMIIB, or NMMIIC siRNAs and on day 4 post-transfection were double-immunolabeled for myosin II heavy chains (green) and E-cadherin (red). Control cells and cells with the myosin II isoforms knock-down show predominant localization of E-cadherin at areas of cell-cell contact which is characteristic of normal AJs (arrows). (2.68 MB TIF) [file pone.0000658.s001.tif]

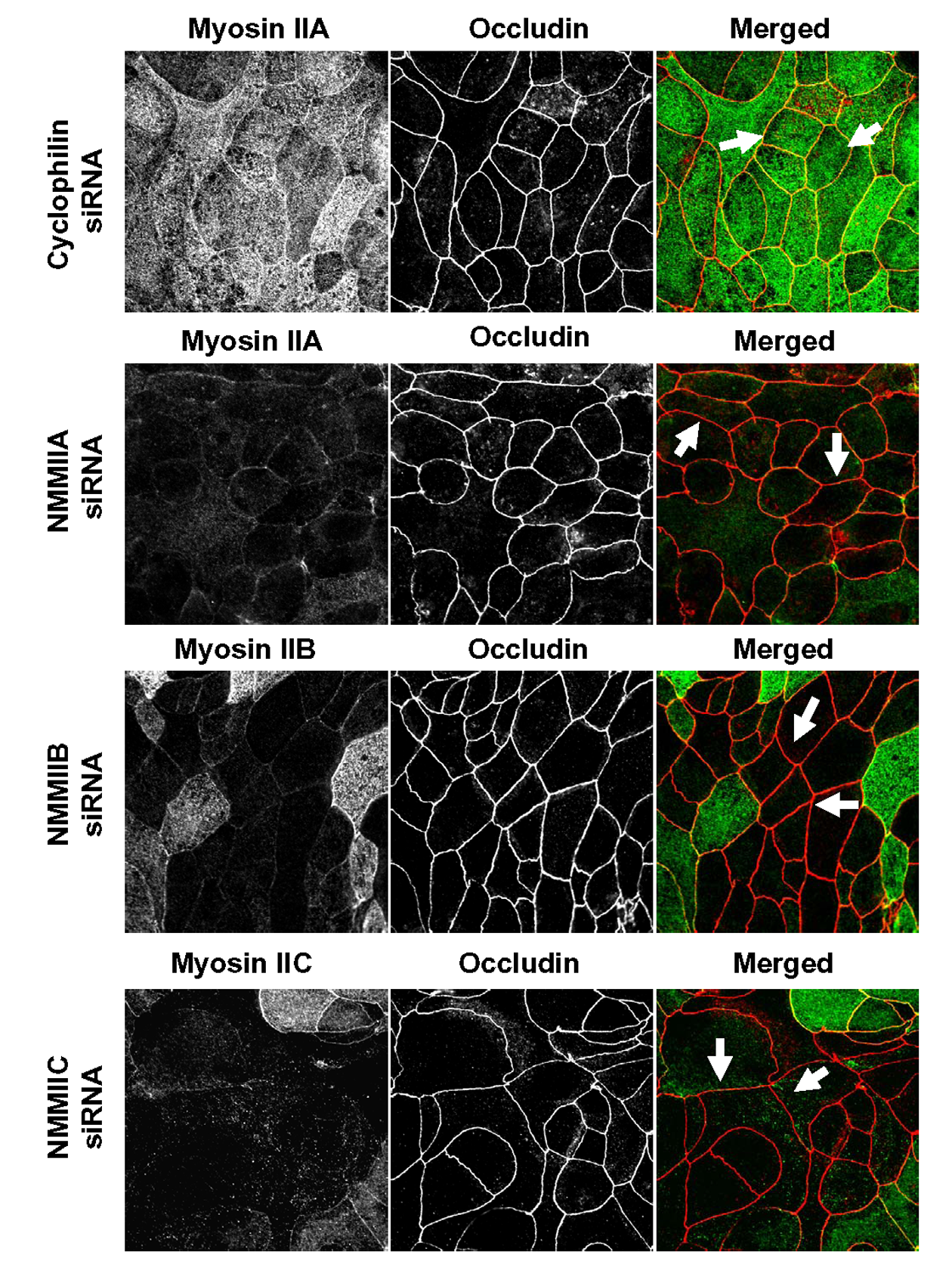

Supplement: Figure S2 — siRNA-mediated knock-down of individual NMMII isoforms does not affect the morphology of mature epithelial TJs. SK-CO15 cells were transfected with either control, NMMIIA, NMMIIB, or NMMIIC siRNAs and on day 4 post-transfection were double-immunolabeled for myosin II heavy chains (green) and occludin (red). Similar to control monolayers, cells with NMMIIA, NMMIIB, and NMMIIC knock-down show a ‘chicken wire’ labeling pattern for occludin (arrows) indicative of normal TJs. (2.85 MB TIF) [file pone.0000658.s002.tif]

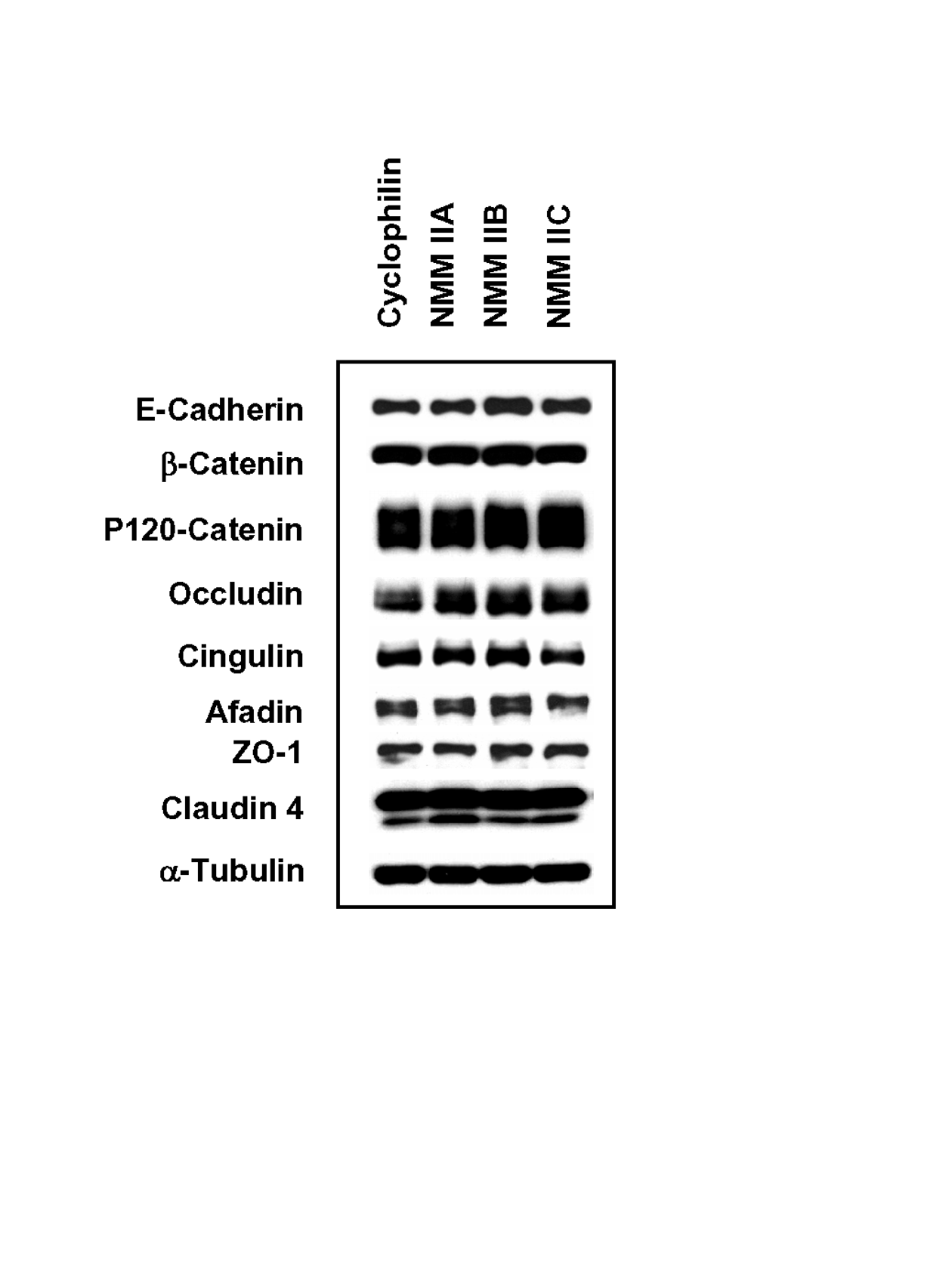

Supplement: Figure S3 — siRNA-mediated down-regulation of NMMII isoforms has no effect on the expression of major AJ/TJ proteins. SK-CO15 cells were transfected with either control, NMMIIA, NMMIIB, or NMMIIC siRNAs and 4 days later were analyzed for expression of different AJ and TJ proteins by Western blotting. Note that expression of junctional proteins was not affected by down-regulation of individual NMMII isoforms. (0.34 MB TIF) [file pone.0000658.s003.tif]
